# A test for comparing two groups of samples when analyzing multiple omics profiles

**DOI:** 10.1186/1471-2105-15-236

**Published:** 2014-07-08

**Authors:** Nimisha Chaturvedi, Jelle J Goeman, Judith M Boer, Wessel N van Wieringen, Renée X de Menezes

**Affiliations:** 1 Epidemiology and Biostatistics, VU University Medical Center, Amsterdam, The Netherlands; 2 Department of Medical Statistics and Bioinformatics, Leiden University Medical Center, Leiden, The Netherlands; 3 Department of Pediatric Oncology/Hematology, Erasmus MC-Sophia Children’s Hospital, Rotterdam, The Netherlands; 4 Netherlands Bioinformatics Center, Nijmegen, The Netherlands; 5 Department of Mathematics, VU University Amsterdam, Amsterdam, The Netherlands; 6 Biostatistics, Department for Health Evidence, Radboud University Medical Center, Nijmegen, The Netherlands

**Keywords:** Group effect, Joint analysis, Penalized regression

## Abstract

**Background:**

A number of statistical models has been proposed for studying the association between gene expression and copy number data in integrated analysis. The next step is to compare association patterns between different groups of samples.

**Results:**

We propose a method, named dSIM, to find differences in association between copy number and gene expression, when comparing two groups of samples. Firstly, we use ridge regression to correct for the baseline associations between copy number and gene expression. Secondly, the global test is applied to the corrected data in order to find differences in association patterns between two groups of samples. We show that dSIM detects differences even in small genomic regions in a simulation study. We also apply dSIM to two publicly available breast cancer datasets and identify chromosome arms where copy number led gene expression regulation differs between positive and negative estrogen receptor samples. In spite of differing genomic coverage, some selected arms are identified in both datasets.

**Conclusion:**

We developed a flexible and robust method for studying association differences between two groups of samples while integrating genomic data from different platforms. dSIM can be used with most types of microarray/sequencing data, including methylation and microRNA expression. The method is implemented in R and will be made part of the BioConductor package SIM.

## Background

Many experimental studies produce multiple types of molecular profiles per sample to help understand if, and how, different molecular levels influence each other. This has led to a growing interest in methods for analyzing multiple high-dimensional datasets together. As a result, a number of methods have been developed in recent years for studying the association patterns between high-dimensional datasets, i.e. performing integrated genomic analyses [1-6]. In a study where DNA copy number and gene expression profiles are available for all samples, there is often interest in questions such as which copy number changes effectively affect gene expression levels, as well as which genes have their expression levels regulated by copy number changes. Both questions can be answered by a method such as the one described in [7]. This method is characterized by testing for the association between one variable in one dataset, say copy number at a fixed locus, and a set of variables in the other dataset, such as the expression levels of genes around the locus. This approach is implemented in R and available through Bioconductor as the package SIM.

Studying these association patterns using integration analysis evokes another very important but yet overlooked question: Do these association patterns differ between groups of samples? Consider for example the case where gene expression regulation is studied on the basis of genomic copy number changes. In such studies, there is often interest in comparing gene-dosage led gene expression variation between groups of samples. Furthermore, if there are clinical variables defining the groups of samples, there is interest in finding group-specific associations between copy number and gene expression.

In this paper, we propose a method, named dSIM, to find these association differences between two groups of samples, while using an extension of the model described in [7]. It first corrects for the baseline association present for both groups using ridge regression [8]. Testing is done using the global test [9,10] on the residual associations, to check if they differ between the groups. The final p-values are calculated using permutation testing. The various steps involved in the method are discussed in more detail in the methods section (section ‘Methods’). To demonstrate its performance, we applied dSIM to several simulated datasets (section ‘Simulation study’) and to two publicly available breast cancer datasets (section ‘Application to breast cancer data’), where we compare samples on the basis of their estrogen receptor status. Throughout this paper we use examples based upon copy number and gene expression datasets, but this method can be applied to other types of genomic data as well.

## Methods

### Motivation

We first give a brief overview of the model and method of SIM [7] before describing our extension to that method. Suppose there is interest in finding regions where copy number variation regulates gene expression. Then, if the copy number data corresponding to a single copy number probe **y** with *n* observations is **y** = (*y*_1_,*y*_2_,…,*y*_*n*_)^⊤^, the regression model can then be written as

(1) $$\begin{array}{l} E(y_{j})=\alpha +\overset{p}{\underset{k=1}{\sum }}\beta_{k}X_{\text{jk}},\;j=1,\ldots ,n, \end{array}$$

*X*_*jk*_ is the gene expression measured for *j*^*th*^ sample and *k*^*th*^ probe, and *α* is the intercept. Here, *β*_*k*_ is the coefficient value for *k*^*th*^ gene expression probe; it explains the association patterns between copy number probe **y** and gene expression data {*X*_*jk*_,*j*=1,…,*n*;*k*=1,…,*p*}. For testing the copy number and gene expression associations, the global test [9,10] is used. The global test tests the hypothesis {*H*_0_:*β*_*k*_ = 0,*k* = 1,…,*p*} in high-dimensional data by using a random effects model context, where it is assumed that the coefficients {*β*_*k*_} come from a normal distribution with mean 0 and variance *ω*^2^. Instead of testing *H*_0_:*β*_*k*_ = 0, the global test [9,10] considers the null hypothesis *H*_0_:*ω*^2^ = 0 against the alternative *H*_1_:*ω*^2^>0.

The main objective of this work is to test for differences in association between copy number and gene expression data, when the samples are grouped according to some binary grouping variable (for example estrogen receptor status in the case of breast cancer). In other words, we would like to find regions where copy number variation regulates gene expression differentially between two groups. One possible way of doing this would be to divide the datasets into two groups and to fit the model given in (1) for these two groups separately. However, this idea has three serious drawbacks. First, analyzing the two subsets separately results into two distinct sets of p-values, that should be combined and interpreted. Second, it is inefficient as the power of finding associations is reduced by considering a subset of samples at a time. The third issue ignored by analyzing the datasets separately like this is the baseline association between copy number and gene expression. Association patterns common to both groups should be removed before we start looking at the differences between them.

Our new approach dSIM extends the model given in (1) as follows. It starts by correcting for the baseline associations and then works with the corrected data to directly test for differential association patterns. For the sake of simplicity, we describe the model in the following subsections by using one copy number probe at a time. In practice and in the examples shown in this paper, it is applied to all copy number probes in a similar way.

### Correcting for the baseline association

For removing copy number and gene expression associations common to both groups, we start by fitting the model given in (1) on all samples together. The issue of (*p*≫*n*) is dealt with by fitting (1) using ridge regression [8]. It is obvious that the ridge regression fit requires estimation of a tuning parameter *λ*. We do this by leave-one-out cross-validation. For each *λ* value, the dataset is divided into a test set containing a single sample *i* from the original sample set, and a training set containing all remaining samples with *j* = 1,…,*n*,*j*≠*i*. The ridge regression model is fitted and the coefficients are estimated on the training dataset. Note that the coefficients estimated here are obtained without introducing the group effect. These estimated coefficients are then used to predict the test sample as

(2) $$\begin{array}{l} E\left(y_{i}^{P}\right)=\alpha +\overset{p}{\underset{k=1}{\sum }}{\hat{\beta }}_{k}^{-i}X_{\text{ik}},\;i=1,\ldots ,\mathrm{n.} \end{array}$$

where ${\hat{\beta }}_{k}^{-i}$ is the estimated coefficient for the *k*^*th*^ gene expression probe, obtained after leaving the *i*^*th*^ sample out from the training set. This is repeated such that each sample is used once as the test set. Once this is done for all *λ* values, the *λ*^∗^ leading to the set {*y*_*i*_^*P*∗^} which maximizes the cross-validated likelihood is chosen. The predictions {*y*_*i*_^*P*∗^} obtained during cross validation for all samples using that *λ*^∗^ value are then used to get the leave one out cross-validated residuals as

(3) $$\begin{array}{l} R_{j}=y_{j}-y_{j}^{P*},\;j=1,\ldots ,\mathrm{n.} \end{array}$$

This approach helps in choosing a *λ* that does not overfit the data. The residuals {*R*_*j*_,*j* = 1,…,*n*} now represent the copy number data corrected for association with gene expression over all samples regardless of the group.

### Testing for the group effect

The cross validated residuals {*R*_*j*_} from (3) are now used for testing the association differences between the two groups of samples. For performing the test, first we write a linear model with the residuals as dependent data. This model holds for all samples together and is given as

(4) $$\begin{array}{l} R_{j}=\delta c_{j}+\overset{p}{\underset{k=1}{\sum }}\gamma_{k}M_{\text{jk}}+\varepsilon_{j},\;j=1,\ldots ,\mathrm{n.} \end{array}$$

In this model, *δ* acts as the intercept and *δc*_*j*_ represents the baseline shift in association between copy number and gene expression for group 2 when compared to group 1. Here, *c*_*j*_∈**c**, where **c**, the factor defining the groups, can be written as **c** = (*c*_1_,*c*_2_,…,*c*_*n*_)^⊤^. Now, since {*R*_*j*_} represents average residual effects over all samples, it is important to parameterize **c** in such a way that **1**^⊤^**c** = 0. Here **1** is a column vector of ones with length *n*. Therefore, for all examples in the paper, we define $c_{j}=\frac{n_{G2}}{n}$ if the *j*^*th*^ sample belongs to group 1 and $c_{j}=-\frac{n_{G1}}{n}$ if the *j*^*th*^ sample belongs to group 2, where *c*_*j*_ are values in **c** and *n*_*G*1_, *n*_*G*2_ are the number of samples for group 1 and group 2 respectively. The value *M*_*jk*_ is the gene expression data for the *j*^*th*^ sample and *k*^*th*^ probe with grouping effect in it. It can be defined, in words, as an gene × group interaction term or *M*_*jk*_ = *X*_*jk*_*c*_*j*_, where *X*_*jk*_ is the gene expression measured for *j*^*th*^ sample and *k*^*th*^ probe and *c*_*j*_ is the corresponding group variable. The error term in the model is denoted by *ε*_*j*_.

The effect of interest in model (4) is represented by the parameters {*γ*_*k*_,*k* = 1,…,*p*}, which account for the difference in association between the copy number data and gene expression data between the two groups. Similar to the {*β*_*k*_} coefficients, we here assume that {*γ*_*k*_} come from a normal distribution with mean 0 and variance *θ*^2^. Our approach is to then test the variance for the distribution of these {*γ*_*k*_} values with, *H*_0_:*θ*^2^ = 0 against the alternative *H*_1_:*θ*^2^>0. This is done by using the global test [9,10]. More information on the test statistic used is given in the Additional file 1: section 2. The distribution of the test statistic under the null hypothesis *H*_0_:*θ*^2^ = 0 is not known because of the ridge regression step. Hence, we propose to use permutation of the independent data sample labels to yield p-values.

### Permuting the sample labels

Permutation resampling can be used to estimate p-values while avoiding parametric assumptions about the joint distribution of the test statistic. In the microarray setting, the joint distribution under the complete null hypothesis of the test statistic can be estimated by permuting the rows of the matrix **X**, where **X** is the *n*×*p* gene expression data matrix with samples as rows and genes as columns. For this, we first permute the sample labels *B* times. The baseline association is corrected, residual values are obtained, and the global test [9,10] is done for every permuted dataset. For each permutation we get a global test statistic $T_{i}^{*}$, or, as in our case, 1-1 transformation of it such as the asymptotic global test p-value $P_{i}^{*}$, leading to a set of permutation based values $\left\{T_{i}^{*},\;\;i\;=\;1,\ldots ,B\right\}$ or $\left\{P_{i}^{*},\;\;i\;=\;1,\ldots ,B\right\}$.

As described in subsection ‘Correcting for the baseline association’, while fitting the regression model and calculating the residuals using ridge regression, one needs to optimize the tuning parameter *λ*. This ridge regression step given in (2) does not involve the grouping factor **c**. Moreover, permuting the sample label of **X** retains the correlation structure, distributional characteristics exhibited by the genomic data matrix **X** and the ill-conditionedness of **X**^⊤^**X**. Since *λ* depends on **X**^⊤^**X**, we can use the same optimized *λ* value and hence, the same residuals for each permutation. Permuting the sample labels avoids the optimization of *λ* parameter for every permutation, making the method computationally less intensive.

The steps involved in the method described in the previous subsections as well as this one are done for a single copy number probe at a time. These are then repeated for all *m* copy number probes which gives us a matrix of global test p-values, *P*^∗^, with *m* rows and (*B*+1) columns. The first column of this matrix consist of the observed global test p-values for all probes, obtained for the observed data (subsection ‘Testing for the group effect’), whilst remaining columns represent permuted global test p-values obtained after permuting the sample labels.

### Multiple testing correction

To exploit the dependence structure of the permuted dSIM p-values, we use Meinshausen’s procedure for multiple testing correction [11]. For general sets of hypotheses *R*, Meinshausen’s permutation based method makes false discovery proportion (FDP) confidence statements of the form P(o(R)≤ō(R))≥1-α by finding critical values. Here *o*(*R*) is the proportion of type I errors for the set *R* in hypothesis testing and ō(R) is the estimate of this proportion [11,12]. We apply this procedure on the matrix of permuted global test p-values *P*^∗^, which gives us a single vector of length *m*, containing the multiple testing corrected dSIM p-values.

## Results

### Simulation study

We perform various simulations to study the properties of dSIM. For this, we start with generating the copy number data and gene expression data, both of them with same dimensions. The copy number probes are assumed to have similar distribution across the two groups and the gene expression data is generated per group, as a function of copy number. In our case, we consider copy number as the dependent data and use Meinshausen’s approach for multiple testing correction. More details on the simulation setup are given in the Additional file 1.

#### *dSIM detects correct regions*

**Figure 1:**
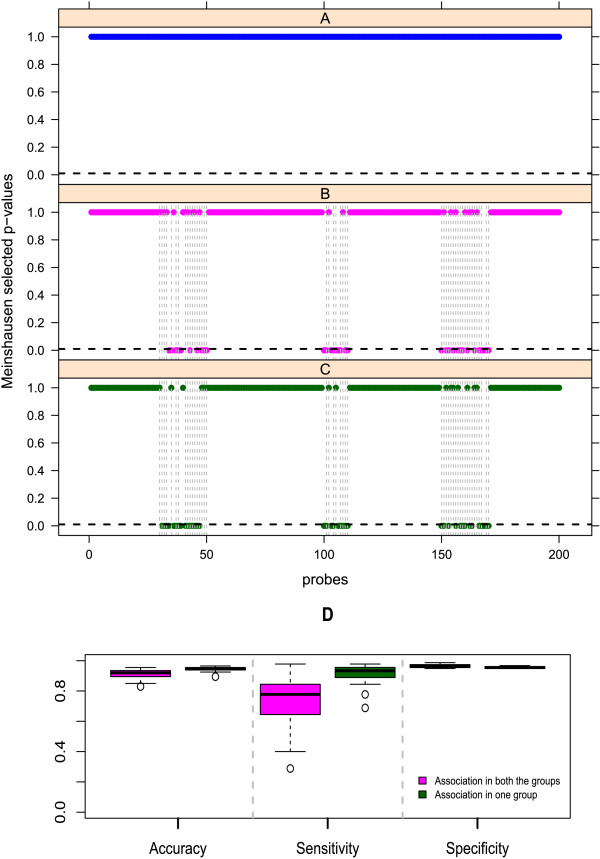
**dSIM detects correct regions.(A)**: 1 of 50 runs for simulation study 1 with no difference in association patterns. **(B)**: 1 of 50 runs for simulation study 2 with different levels of association effects between the groups. **(C)**: 1 of 50 runs for simulation study 3 with association patterns for one group while no associations between copy number and gene expression for the other group. Significant p-values, selected by Meinshausen’s procedure at significance level 0.01 (horizontal black dotted line), are the ones close to 0. The regions with differential associations in each of the three cases, are marked by gray dotted bars. **(D)**: This figure summarizes the accuracy, sensitivity and specificity for the 50 simulation runs.

This simulation study tests if dSIM is able to detect desired regions of differential associations. It also tests the accuracy of dSIM by studying the number of false positives in the scenario when there is no differential association between groups of samples. We test dSIM in three different scenarios. In each scenario, we vary the type of association present between copy number data matrix **Y** and the two different groups of gene expression data **X** as follows: ***Scenario 1*** For this scenario, copy number and gene expression show similar association patterns for both groups of samples. Hence, no significant association differences between the two groups are expected. Under this scenario, we test the specificity of dSIM. ***Scenario 2*** In this scenario, copy number and gene expression show different association patterns between groups of samples. Hence, significant association differences between two groups should be detected. ***Scenario 3*** This scenario has association patterns between copy number and gene expression for one group of samples only, so here again differences should be detected. This scenario is similar to the second scenario in the sense that there are association differences present between groups of samples.For scenario 1, no probes are selected, as expected (Figure 1A). For scenario 2, most probes in regions affected are detected (Figure 1B), although there are some false negatives. Results of scenario 3 are similar to those of scenario 2 (Figure 1C). For each scenario, 50 datasets were simulated, and the accuracy of dSIM was calculated based on those 50 runs. Figure 1D (for scenarios 2 and 3) shows that dSIM has overall good accuracy and specificity, although it sometimes has false negatives, lowering the sensitivity.

#### *dSIM corrects for baseline effect*

The validity of the results obtained from dSIM is based on how well we correct for the baseline association. The model uses the simple structure of explaining copy number aberrations on the basis of gene expression variations. One of the factors that might differ between these two groups is the distribution of copy number variations or gene expression intensities.

If the method does not correct for baseline effects, then our results will be biased, resulting in possible false positives. To test if dSIM really corrects for the baseline association, we perform 4 simulation studies. These four simulation studies are divided under two cases as follows, **Case 1:** The copy number and gene expression data for both groups of samples were generated with similar association patterns. Hence, there is no association differences between groups of samples. **Case 2:** The copy number and gene expression data for both groups of samples were generated with different association patterns. Hence, there is significant association differences between groups of samples.

Under each of these two cases we perform two simulation studies which can be described as follows, *Case 1.1 and Case 2.1*: The distribution of copy number aberrations are kept exactly the same for the two groups of samples. This makes sure there are no distributional differences between the two groups for copy number aberrations. *Case 1.2 and Case 2.2*: The distribution of copy number aberrations for one group of samples are made to differ from the other group. This is achieved by using different means for generating the copy number aberrations for the two groups of samples.

**Figure 2:**
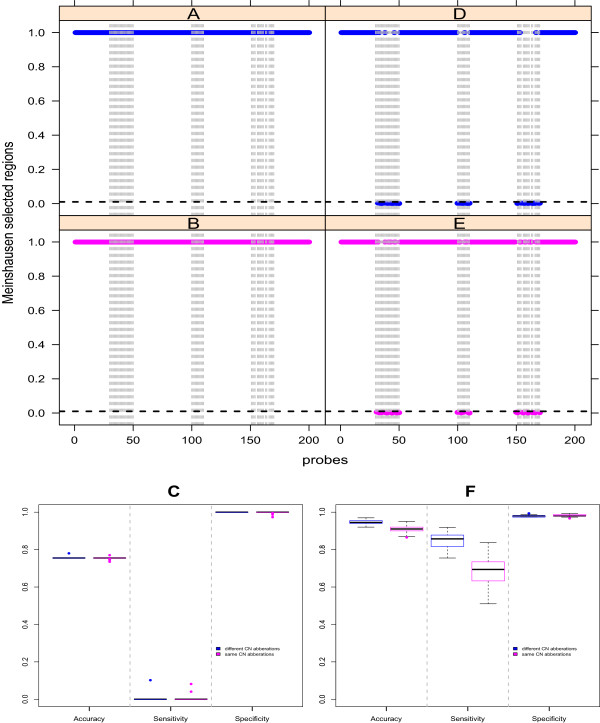
**dSIM corrects for baseline effect.(A)**: 1 of 50 runs for case 1.1 with no differential association and similar distribution of copy number aberrations between the groups of samples. **(B)**: 1 of 50 runs for case 1.2 with no differential association and different distribution of copy number aberrations between the groups of samples. **(C)**: Accuracy, sensitivity and specificity for 50 runs, for the two simulation studies under case 1. **(D)**: 1 of 50 runs for case 2.1 with differential association and similar distribution of copy number aberrations between the groups of samples. **(E)**: 1 of 50 runs for case 2.2 with differential association and different distribution of copy number aberrations between the groups of samples. **(F)**: Accuracy, sensitivity and specificity for 50 runs, for the two simulation studies under case 2. Significant p-values, selected by Meinshausen’s procedure at significance level 0.01 (horizontal black dotted line), are the ones close to 0. The regions with differential associations in each of the four figures, are marked by gray dotted bars.

All the other parameters are exactly the same between the cases, including the number of samples, number of genes etc. Each study was then run 50 times and dSIM was applied on these 50 simulated datasets for the simulations.Figure 2 shows the regions picked up in one of the runs for all simulation studies. For case 1 (Figure 2A and 2B), dSIM detects nothing as significant with similar or different copy number aberrations as there are no differential associations. The sensitivities and accuracies (Figure 2C) are comparable for datasets with similar copy number aberrations and different copy number aberrations. Similarly, for case 2 (Figure 2D and 2E) the desired regions with differential associations are detected by dSIM in both sub cases. The accuracies and sensitivities (Figure 2F) in this case are also similar for datasets with similar and different copy number aberrations between groups of samples. These results show that dSIM correctly adjusts for the baseline effect. It detects significant results only when there is differential association between two groups, irrespective of their copy number distributions.

#### Sensitivity of dSIM towards changes in lambda values

Our results depend upon the ridge penalty parameter *λ*, which is estimated using cross-validation. We will now study the sensitivity of dSIM towards the variation of these *λ* values.

Firstly, we study the variation of *λ* value within and across different cases. For this, we conduct four different simulation studies. In every simulation study, a certain parameter that affects the value of lambda (number of probes, association differences, signal to noise ratio) is changed. Each study is then run 50 times, producing 50 simulated datasets from the same setup. The data points for the matrix **X** change between these 50 runs, but the number of probes and the regions showing differential association between the the two groups remain exactly the same. For every run, the *λ* value is estimated using the method described in section ‘Correcting for the baseline association’. This makes sure that while the value of *λ* might vary a lot across the four simulations, intra-study variations would be minimal. The ROC curves for these simulation studies are given in Additional file 1: Figure S1 (A,B,C,D). Each graph depicts an individual simulation study with 50 ROC curves for each run of the setup. From the graphs we can see that, for a given setup, all 50 ROC curves are tightly bound together with very little or almost no variation. This suggests that the true positive rate of dSIM remains the same even if *λ* displays some variations.

Secondly, we study the effect of changing *λ* values over the dSIM p-values. For this we perform a simulation study where only some simulated copy number probes show differential association with gene expression data. For each probe, the optimum lambda value (*λ*_*opt*_) is obtained and a range of 50 *λ* values around *λ*_*opt*_ is generated. For each of these *λ* values, Meinshausen-selected dSIM p-value is calculated, resulting in a vector of 50 p-values for each probe. These p-values are then plotted against the corresponding *λ* value for some chosen probes. From Additional file 1: Figure S1E and 1F, we can see that the Meinshausen selected dSIM p-values exhibit a similar trend over a wide range of *λ* values. The probes that exhibit differential association between groups of samples are selected as significant irrespective of the *λ* values. The probes with no association, on the other hand, are never selected by dSIM and Meinshausen’s method, irrespective of the changes in *λ*_*opt*_. This shows that dSIM is capable of identifying the probes with differential association and maintaining the true positive rate, even with small variations in the value of *λ*_*opt*_.

#### Effect of small sample size on separate analysis and dSIM

To demonstrate that separate analysis suffers from differences in sample sizes, we performed a small simulation study. In this study we simulated 50 datasets with 45 samples and 100 probes in each. Out of these 45 samples, 30 were assigned to group 1 and 15 to group 2, so that to have one larger and one smaller group of samples. All the other parameters between the two groups of samples are kept exactly the same, including the region of copy number aberrations. The samples in both groups show similar type of associations with between copy number and gene expression data. Hence, there is no differential association between the two groups of samples.

We started with performing a separate analysis of these 50 datasets, using SIM. The multiple tested corrected p-values obtained from separate analysis, for one of the 50 simulated datasets, are shown in Additional file 1: Figure S2A. From the figure, we can see that although the associations between gene expression and copy number are exactly the same for both groups, separate analysis is more sensitive for the larger group of samples. These separate analysis results give a specious indication that there is differential association between the two groups of samples. On the other hand, dSIM results, in Additional file 1: Figure S2B, are in line with the simulation setup with no significant detection of differences in associations. This shows that separate analysis gets influenced by the sample size, specially when it is small. The overall sensitivity of separate analysis and dSIM, for 50 simulated datasets, is shown in figure Additional file 1: Figure S2C. It can be clearly seen that the separate analysis is more sensitive towards larger number of samples. On the other hand, the overall sensitivity and specificity for dSIM is not affected by the sample size differences.

### Application to breast cancer data

#### Datasets

We apply dSIM to two publicly available breast cancer datasets, namely, the TCGA dataset and the NKI dataset [2]. Both datasets are very different when considering the platforms, genomic coverage, and the sample sizes used. The TCGA data (166 samples) consist of copy number (SNP 6.0) for 29101 locations (segmented data) and gene expression (Agilent) for 74895 probes. In the NKI dataset, the arrayCGH (BAC arrays) data and the gene expression data (Agilent) have both 18,184 probes and 68 samples.

We considered estrogen receptor (ER) status of the samples as the grouping variable. The TCGA dataset consists of 134 ER-positive samples and 32 ER-negative, while the NKI data consists of 43 ER-positive and 25 ER-negative samples. ER status is considered an important prognostic and a predictor factor for endocrine response in breast cancer. It has been shown to have strong association with the prognosis of the disease and exhibits strong associations with distinct gene expression patterns [13-15]. This makes estrogen receptor status an interesting grouping variable when studying differential associations in the TCGA and NKI breast cancer datasets.

#### Separate analysis using SIM

We start by simply analyzing the NKI and TCGA datasets by fitting model (1) per group, ER-positive and ER-negative. We report the results through whole genome plots for both groups, while controlling the FDR at 5%. The R package SIM was used to perform these analyses. The results from the separate analyses are then compared with the results from the application of dSIM to the NKI and TCGA datasets.

The selected probes for the two groups of samples, obtained from separate analyses of the NKI dataset, are shown in Additional file 1: Figure S3. It can be seen from the figures that a lot of associations present for the larger ER-positive (43) group, are not there for the smaller ER-negative (25) group, for example chromosome arm 1q and chromosome arm 3p. These different results for the groups might be due to sample size difference between the two groups. On the other hand, many associations on chromosome arms 12p and 20p are specific to ER-negative group of samples and are not detected for the larger ER-positive group. Apart from group-specific effects, there are chromosome arms, such as 17q, where separate analyses detects effects in both groups, and chromosome arm 7q, where not many effects are seen for any of the two groups.

Similar results can be seen from the separate analysis of TCGA dataset also (Additional file 1: Figure S4). Most of the associations detected for the larger ER-positive group are not detected for the smaller ER-negative group. Analyzing the two groups separately produces two distinct sets of p-values. These p-values are useful when answering questions about group-specific associations between copy number aberration and gene expression levels. However, we cannot say anything about the differences between these association patterns without an additional step of combining these p-values and measuring the error. This makes the separate analyses more complicated and less interpretable when it comes to studying differential association patterns between two groups of samples. In addition, any combination of these two separate analysis results will have power limited by the smallest sample size, which in this case is only a quarter of the size of the biggest groups.

#### Joint analysis using dSIM

We now apply dSIM on both NKI and TCGA datasets. Since we consider copy number as the dependent data, Meinshausen’s procedure is used for multiple testing correction while controlling the significance level at 0.1.

**Figure 3:**
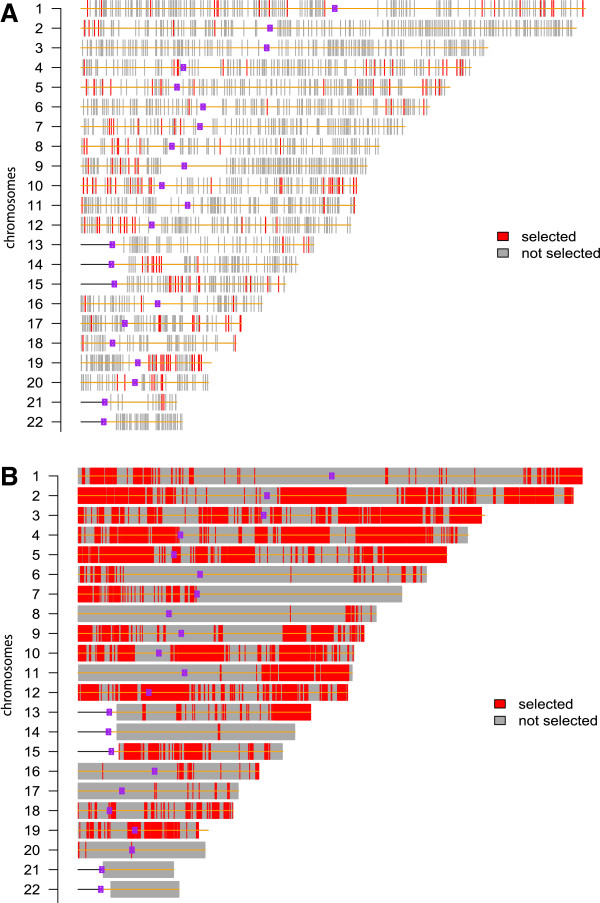
**dSIM selections for NKI and TCGA datasets.(A)**: Probes selected by dSIM for NKI (68 samples) breast cancer data. **(B)**: Probes selected by dSIM for TCGA (166 samples) breast cancer data. Meinshausen’s procedure is used for multiple testing correction while controlling the significance level at 0.1. Chromosomes are represented by horizontal bars. Each vertical bar represents one copy number probe, with color of the bar indicating the test result: red, selected; gray, not selected.

As described in section ‘Methods’, dSIM takes all samples together while testing for differential associations, thus, avoiding the loss of power of separate analyses per group. From Figure 3, it can be seen that significant probes were identified in partially overlapping regions in the two datasets despite the larger power yielded by the TCGA data due to having twice as many samples as the NKI data. Some examples are chromosome arm 1q, 7q, 12p, and 17q. In the case of chromosome arm 1q, the difference seems to be due to a relatively larger gene dosage affecting gene expression levels in the ER-positive group (Additional file 1: Figure S5 for NKI data). Although the difference in copy number is subtle, it is enough to motivate differential associations. The findings from dSIM for chromosome arm 1q are in line with the results obtained from the separate analyses. This suggests that the absence of effects for ER-negative group, in contrast with effects for ER-positive group, may not be simply due to sample size difference between the two groups.

Another chromosome arm in the NKI data where separate analyses detect strong association patterns for ER-positive group of samples, but very few for ER-negative groups of samples, is 3p. However for this chromosome arm, dSIM does not detect significant probes for the NKI dataset (Figure 3). If we examine the empirical p-value distribution yielded by SIM (Additional file 1: Figure S6), we note that p-values do not follow a uniform distribution, suggesting that there are associations between copy number and gene expression in both groups. However, due to the smaller sample size in the ER-negative group, very few probes are selected at 5% FDR. Thus, the differences in significant probes found for ER-positive and ER-negative group are likely due to lack of power in the smaller ER-negative group, in line with the dSIM results.

In contrast with 1q, separate analyses of the two groups of samples for chromosome arm 12p identified most of the significant probes in the smaller group of samples with ER-negative. When dSIM is applied to 12p for NKI and TCGA datasets, similar regions are identified (Additional file 1: Figure S7), confirming the differential association patterns suggested by the separate analyses.Some other examples of chromosome arms where separate analyses results correspond with dSIM results are 17q and 7q. For 17q, dSIM detects significant association differences between the two groups in both datasets (Figure 3). This supports the separate analyses result for 17q where it detects association effects in both groups, though more strongly in the larger ER-positive group than in ER-negative group. Similarly, for chromosome arm 7q, separate analyses results show almost no associations between copy number aberrations and gene expression levels for both groups. Correctly, dSIM also does not find differences in associations.

#### A closer look at 11q13

An interesting chromosome region to analyze is 11q13. A recent study shows that the amplification of chromosomal region 11q13, including the *CCND*1 gene is associated significantly with ER-positive breast tumors [16]. We analyzed this chromosome region using SIM and dSIM on the larger TCGA dataset to see if copy number driven gene expression differs between ER-positive and ER-negative groups of samples.

From the Additional file 1: Figure S4, we can see that separate analysis of ER-positive and ER-negative group of samples, using SIM, suggests differential associations by detecting a lot of associations in the larger ER-positive group and no associations in the ER-negative group. However, as the ER-negative group involves a much smaller sample size than the ER-positive group, we cannot be sure that these differences are not due to differences in power. For refining the results further, we reduced the window size to 2 Mb (including the *CCND*1 gene) so as to focus just on 11q13 and re-analyzed the datasets using SIM. The raw p-value distributions are shown in Additional file 1: Figure S8A, where it is evident that the larger ER-positive group has strong associations between copy number and gene expression levels. On the other hand, the smaller ER-negative group of samples show almost no association between copy number and gene expression. These separate analysis results suggest that 11q13 associations are indeed specific to ER-positive subtype and hence suggests differential association between ER-positive and ER-negative group of samples. However, when we analyzed the same region of 11q13 using dSIM, it detected no significant differential associations between the two groups of samples (Additional file 1: Figure S8B). This motivated us to explore the datasets the underlying association patterns in more details.

Firstly, we fitted a ridge regression between each copy number probe and all gene expression probes in 11q13. We then found that coefficients with the largest ridge estimates tended to be the same for ER-positive and ER-negative groups of samples, for those copy number probes with significant association with all gene expression probes, as given by SIM. Secondly, we looked at the SIM results for these copy number probes in more detail, by studying the individual (standardized) influences of each gene expression probe on the global test. The scatter plot of global test z-scores for ER-positive and ER-negative groups of samples are shown in Additional file 1: Figure S9 for a chosen copy number probe. From Additional file 1: Figure S9, we can see that both in ER-positive and ER-negative groups of samples same probes drive the SIM results. From these results, it is evident that gene expression probes with the largest influences are the same, for ER-positive and ER-negative groups of samples, confirming the ridge regression results. This led us to conclude that ER-positive and ER-negative groups of samples display the same copy number-led regulation of gene expression on 11q13, in agreement with dSIM results. The lack of power due to small sample size is also one of the reasons why SIM suggests differential association, whilst dSIM does not. However, here similar underlying association patterns in ER-positive and ER-negative groups of samples lead to dSIM not detecting differences in association between groups.

#### Biological interpretation

From the biological viewpoint, it is important to check if the genes detected by dSIM are known to be associated with breast cancer and more, importantly, estrogen receptor status. Literature review brings up important breast cancer-associated genes located in regions detected by dSIM. Here we focus on chromosome arm 12p of the NKI dataset, where tumors in the ER-negative group showed more frequent gains compared with the ER-positive group, and several regions of differential association were detected. We selected the five most differentially associated copy number probes on 12p (Additional file 1: Figure S10) and for each of those, four gene expression probes were selected based on association priority (global test p-values signifying their association with the selected copy number probe) and proximity (closest to selected copy number probe based on their genomic locations). More information on the selection criterion and the list of copy number probes along with the selected gene expression probes is given in the Additional file 1: Table S1.

In the first region, from 1 to 2 Mb, one copy number probe was selected, covering a genomic region from 1,855–2,005 kb and including the *ADIPOR*2, *CACNA*2*D*4 and *LRTM*2 genes. The highest associated gene expressions were *ADIPOR*2, which overlaps with the gene expression probe, *RAD*52 and *WNK*1, which are more upstream, and *ITFG*2, which is more downstream (Additional file 1: Figure S11). Loss of heterozygosity of *RAD*52 is related to breast cancer. However, no link is known between *RAD*52 expression and ER status. The adiponectin receptor *ADIPOR*2 was found to be expressed in human breast cancer cells [17-20]. Its ligand adiponectin, an adipocyte-secreted hormone that plays an important role in diabetes and cardiovascular disease, may also be of importance in the development and progression of several malignancies, including breast cancer [21]. A peptide-based adiponectin receptor agonist has been proposed for cancer treatment that restricted proliferation and suppressed growth of human breast cancer xenografts in mice [22]. Since we found *ADIPOR*2 over expressed due to copy number gain in the ER-negative group, these data suggest that ER-negative breast cancer may be a potential target for future adiponectin receptor agonist treatment.

**Figure 4:**
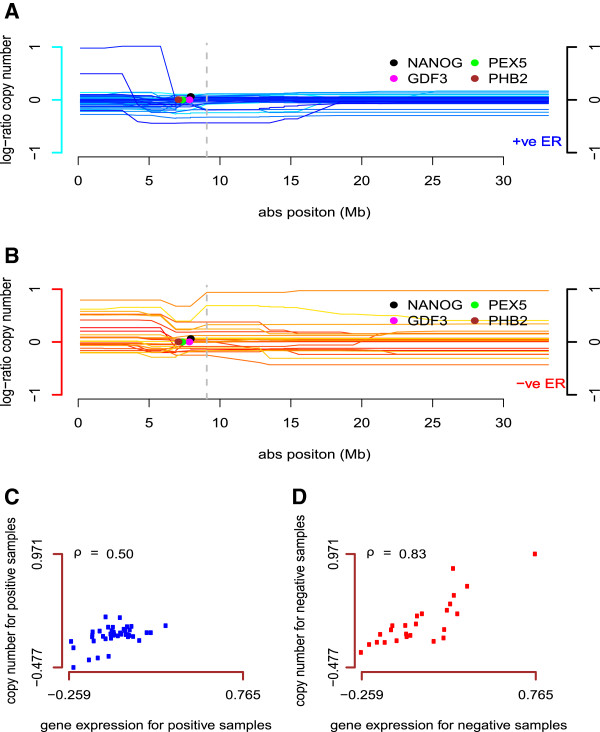
**Data points for selected copy number and gene expression (*PHB*2) probe.(A)**: Significant gene expression probes (colored dots) selected for copy number probe (grey dotted line) on 12p for ER-positive samples (43) in NKI breast cancer data. **(B)**: Significant gene expression probes (colored dots) selected for copy number probe (grey dotted line) on 12p for ER-negative samples (25) in NKI breast cancer data. **(C)**: Association between selected copy number probe and gene expression probe (*PHB*2) data points for all ER-positive samples. **(D)**: Association between selected copy number probe and gene expression probe (*PHB*2) data points for all ER-negative samples.

In the second region, from 8–19 Mb, copy number gains were also more frequently found in the ER-negative group, and the selected gene expression probes showed positive association with copy number in the ER-negative samples (Figure 4, Additional file 1: Figure S12). Among these genes, three have been described in breast cancer. Two embryonic stem cell genes, *GDF*3 and *NANOG*, were shown to be expressed in breast cancer [23]. *GDF*3 was upregulated in 4/24 breast cancers compared to paired normal, and downregulated in 12/24 [24], but no information on ER status of the studied patients was available. Our data suggest that *GDF*3 may be a novel gene whose expression is related to ER status.

Overexpression of *NANOG* was shown to characterize an embryonic stem cell-like signature in breast cancer, associated with high-grade estrogen receptor negative tumors, often of the basal-like subtype, and with poor clinical outcome [25]. Our data suggest that the embryonic stem cell-like signature is associated with increased copy number of 12p that is more frequently found in ER-negative breast cancer. In the same region, *PHB*2 (prohibitin 2), was positively associated with copy number gain in the ER-negative samples (Figure 4C and 4D). *PHB*2 functions as an estrogen receptor (ER)-selective coregulator that potentiates the inhibitory activities of anti-estrogens and represses the activity of estrogens [26]. While upregulation of *PHB*2 might inhibit tumor growth in ER-positive tumors, the biological consequences of upregulation in ER-negative breast cancer are unknown.

It should be noted that in examples where gene expression probe and copy number probe do not overlap with each other, it is important to check for specious associations. It is possible that the dSIM detections are due to co-amplification of copy number probes, for example.

Together, these results show that by testing differential copy number-expression associations between relevant groups of tumors, new hypotheses for tumor biology based on underlying genetic aberrations can be generated.

### Computation time of dSIM

In this subsection we show the approximate computation time of dSIM. To demonstrate how the computation time is effected by size of the gene sets, we ran dSIM on datasets with different number of probes. For all datasets, number of samples in Group 1 and Group 2 were 67 and 16, respectively. Calculation time of dSIM is more affected by the size of the gene set (specially for the outcome data) than by the sample size itself. The reason for this is that dSIM is fitted per outcome gene. Hence, larger the number of genes, higher will be the calculation time. Sample sizes (for dependent and independent datasets) have an effect on the calculation time but it is almost negligible. We ran all analyses using R i386 2.15.2 on an Intel(R) Core2Duo CPU with 3.00 GB of RAM. The run times are given in Table 1.

**Table 1:** Computation time for dSIM for data sets with increasing numbers of copy number probes

**Computation**	**CN probes**	**GE probes**	**Group 1**	**Group 2**
**time (s)**				
18631.36	471	1142	67	16
14148.00	367	1088	67	16
74837.76	850	2935	67	16
55835.08	588	3185	67	16

## Discussion

We proposed a method to compare associations found between two high-dimensional data sets, for two groups of samples. Our method models associations between features in one data set and sets of covariates in the other, thus facilitating the search for markers that are related to a set of features.

Our method uses all data at the same time in the model, thus being less susceptible to small sample sizes in (at least) one of the groups, compared with separate analysis per group. In addition, we consider associations between probes and gene sets. These characteristics make for a powerful method that finds robust differences in associations.

To the best of our knowledge, we are the first to suggest an integrated analysis method for comparing association patterns between two groups of samples. The most closely related method seems to be the one proposed by Artmann et al. [27], as they consider two high-dimensional data sets and a grouping factor. Artmann et al. [27] essentially proposed to first look for differential behavior between features in the two groups of samples, per data set separately. Subsequently, results are combined via meta-analysis. Their proposed method is in the context of microRNA and mRNA analysis, so their meta-analysis involves connecting microRNAs to a set of possible targets. So there is no actual joint analysis of the two data sets, but rather a combination of results of two separate analyses.

Our method does not require that features are differentially valued between the two groups of samples. Indeed, we argue that this is not necessary and, in our view, not even desirable. We have in fact made sure that dSIM results cannot be driven merely by differences on the data distribution of the dependent data between the two sample groups. In our view, such differences in a single data set would not necessarily mean different associations between sample groups, and should not be seen by the method as such either. Furthermore, the lack of differential values does not rule out differential associations, so by considering differential values one actually restricts the results too much.

This makes biological sense. Let us take for example the setup in the TCGA data example. It is entirely possible that DNA copy number behaves in the same way, and displays the same distribution, in both ER-positive and ER-negative groups, and yet are associated differently with gene expression sets in those groups. This could happen due to another molecular mechanism coming into play, say. We feel that an open-minded analysis should be able to pick up those effects.

A unique feature of dSIM is that it corrects for the baseline association before looking for differential associations between groups of samples. Thus, the association effects common to both groups are eliminated, and only those association patterns left over in the residuals are analyzed. In subsection ‘Correcting for the baseline association’, we describe the steps taken by dSIM to correct the baseline association while using ridge regression. The rational behind the usage of ridge penalty is that the metric for the global test statistic is the same as that of ridge. Therefore, the entire method makes use of the same metric.

The residual effects studied by dSIM are relatively small as they are obtained after removing the large association effects during baseline association correction. These effects may weaken further due to overfitting of the ridge penalized model, making them harder to detect. Hence, for detecting these weak effects, it is important to minimize the loss of information during baseline association correction. We ensure this by optimizing the tuning parameter *λ* for ridge penalization using leave-one-out cross-validation. Then the leave-one-out cross-validated predictions, corresponding to the selected *λ* value, are used to get the residuals. Unlike the traditional way of using the fitted predictions, we use the cross-validated predictions, as leaving out a sample during cross-validation makes the estimated coefficients unbiased towards that sample. This, in turn, avoids overfitting of the model when predicting the outcome for the left out sample, hence minimizing the loss while obtaining the residuals.

The fact that we test for a large number of copy number probes simultaneously requires choosing an appropriate multiple testing correction method. As the dSIM p-values are generated using permutation testing, the traditional methods for controlling FWER like Bonferroni, Holm or Hommel are not very useful. Firstly, they are too conservative and secondly, they do not take into account the dependence structure of the data [28,29]. The less stringent FDR control methods such as Benjamini-Hochberg also do not fully exploit the information concerning dependence in the dataset, hence suffering from loss of power. Therefore, we use Meinshausen’s multiple testing correction method which not only takes into account the dependence structure of the permuted p-values, but is also more powerful if the effects are small and spread over a larger region (as in copy number data). Since we consider copy number as dependent data, using Meinshausen’s procedure ensures detection of subtle yet significant effects, like in the case of chromosome arm 1q. Another method that can also be used is the Westfall and Young’s multiple testing correction method [30]. Like Meinshausen’s approach, it also takes into account the dependence structure of the data and is useful for working with permuted p-values when gene expression is considered as the dependent data.

In this paper we focus on analyzing copy number regulated gene expression, where the gene sets are defined on the basis of their genomic locations. The method can be easily extended to analyze other types of genomic data and gene sets as well. For example, there could be interest in finding group-specific interaction effects between microRNA and mRNA expression, while looking at pathway specific genes. It is also possible to invert the model given in (1) to have copy number as independent data and gene expression as dependent data. However, we should indicate that ridge penalty does not exploit the inherit spatial correlation structure found in natural ordering of the copy number probes. In the case where copy number data is independent data, one possible option would be to use fused lasso penalty instead of ridge.

Another interesting extension of this model is to consider more than two groups of samples. The extension involves complex steps as the degrees of freedom goes up from one to *n*_*G*_ - 1, where *n*_*G*_ is the number of groups. As the association patterns are then compared between more that two groups, the number of interaction terms (*γ*) in the model increases. One possible way to compare multiple groups is to use the method proposed in this paper for performing pairwise comparison. This problem is beyond the scope of this paper and will be dealt with elsewhere.

Our method, dSIM, is based on a linear model where we assume that there is a linear relationship between the two genomic datasets. However, we should point out that the non-linear associations present between the datasets may not be detected by dSIM. We also assume the the distribution of the errors to be normal. In cases, with non-normal random errors, one can consider transforming the data before using dSIM. Another assumption made by dSIM is in using the global test for testing the null hypothesis *H*_0_:*θ*^2^ = 0, where *θ*^2^ is the variance of the distribution for {*γ*_*k*_}. The permutation test not only tests for *H*_0_:*θ*^2^ = 0 but also for *δ* = 0, where *δ* is the intercept. The correct size of the test is therefore not guaranteed if *θ*^2^ = 0 but *δ*≠0. However, because we use a global test for the null hypothesis *H*_0_:*θ*^2^ = 0 internally in the permutation, it is unlikely that the permutation procedure has any serious power for *H*_0_:*δ* = 0. In practice, dSIM is not sensitive to those copy number variations that do not affect gene expression levels.

In summary, we developed a method to test for the copy number and gene expression associations differing between two groups of samples. Through several simulation studies, we showed the robustness of dSIM under various conditions. Application of dSIM to the TCGA and NKI breast cancer datasets highlights the importance of having all samples together in the model.

## Conclusion

We propose a novel method for identifying genes that show different expression regulation between two groups. By using all samples together, it can more objectively and effectively find such differences, compared to separate analyses. It can help elucidate differences in gene expression regulation between two groups of samples due to copy number alterations or other (epi) genetic changes.

## Availability of supporting data

The data sets supporting the results of this article are available in the LabArchives repository (https://mynotebook.labarchives.com/share/Supporting\%2520data/My45fDI1MDQyLzMvVHJlZU5vZGUvNDcyNjkzMTc0fDkuOQ==, DOI : 10.6070/H48K771S).

The R scripts for simulation studies and dSIM can be made available upon request.

## Competing interests

The authors declare that they have no competing interests.

## Authors’ contributions

NC implemented the method in R, wrote the manuscript and performed the data analysis. This work uses an original idea by RXM, further developed by RXM, JJG, WvW and NC. RXM and JJG supervised the work. JMB helped with biological interpretation of the results. All authors read and approved of the final manuscript.

## Supplementary Material

Additional file 1 **Supplementary material.** Supplementary document containing details about the simulation study setup, additional figures and tables. Click here for file
