# Supplementary material for: A test for comparing two groups of samples when analyzing multiple omics profiles
Source: BMC Bioinformatics. 2014 Jul 8;15:236. doi: 10.1186/1471-2105-15-236 (PMC4227098; doi:10.1186/1471-2105-15-236)
Supplement: Additional file 1 — Supplementary material. Supplementary document containing details about the simulation study setup, additional figures and tables. [file 1471-2105-15-236-S1.pdf]

Additional fille 1, supplementary material to : A test for comparing two groups of samples when analyzing multiple omics profiles

Nimisha Chaturvedi, Jelle J. Goeman, Judith M. Boer, Wessel N.van Wieringen, Renée X.de Menezes

July 3, 2014

1 Simulation study setup

1.1 Generating copy number data

For each simulation, we first generated a copy number data matrix Y with 100 samples and 200 probes from a normal distribution with mean 0. Out of these 100 samples, 50 samples were assigned to group 1 and 50 to group 2. Out of all samples in group 1, 50 percent randomly chosen samples were assigned values to a predefined stretch of probes from a normal distribution with mean $\neq 0$. Similar step was repeated for group 2 also. This structure simulated a copy number data, where only the selected samples received copy number aberrations in the specified region.

1.2 Generating gene expression data

The number of samples and probes in gene expression data X was kept the same as of copy number data, for all simulations. Out of all 100 samples, only those samples with copy number aberrations could have associations with gene expression data. From the same region where copy number aberrations were assumed, probes were chosen with a given probability for exhibiting associational patterns. For all selected probes gene expression values were generated as,

$$X_{ij}^{G} = \beta^{G} Y_{ij}^{G} + \epsilon, \tag{1}$$

where $j \in S \in G$ with S = a vector containing the indices of the samples with copy number aberrations belonging to $G = G1$ (group 1) or $G = G2$ (group 2). β^{G} is a single coefficient value generated from a normal distribution with mean $\neq 0$. This value remains the same for all i and j. For adding noise to the data, an error value ϵ is added generated from a normal distribution with mean = 0.

For the samples with no aberrations or for the probes which were not selected for showing associational patterns, the values for X were generated from a normal distribution with mean = 0.

1.3 dSIM detects correct regions

This simulation study tests if dSIM is able to detect desired regions of differential associations. It also tests the accuracy of dSIM by studying the number of false positives in the scenario when there is no differential association between groups of samples.

Scenario 1 For this scenario, copy number and gene expression showed similar associational patterns for both groups of samples, hence no significant associational differences between the two groups. Under this scenario, we test the specificity of dSIM. β^{G1} as well as β^{G2} are kept exactly

the same, the value coming from a normal distribution with (mean for β^{G1}) = (mean for β^{G2}) $\neq 0$.
Scenario 2 In this scenario, copy number and gene expression showed associational patterns for both groups of samples but with different intensities. Hence significant associational differences between the two groups are expected. The values for β^{G1} and β^{G2} are generated from normal distribution with (mean for β^{G1}) $\neq$ (mean for β^{G2}) $\neq 0$.
Scenario 3 This scenario show associational patterns between copy number and gene expression for only one group of sample, while for the other group there are no associations present between the two datasets. This scenario is similar to the second scenario in the sense that there are associational differences present between groups of samples. The values for β^{G1} are generated from normal distribution using a certain means and the data values for the aberrated samples in $G1$ were generated using (1). Data values for all other samples in $G1$ and all samples in $G2$ were generated from a normal distribution with mean = 0 and variance = 1.

1.4 dSIM corrects for baseline effect

We performed four simulation studies to test if dSIM is able to correct for baseline association. The aim is to study the effect of copy number distributions on sensitivity and specificity of dSIM. The four simulation studies are divided into two cases and two sub cases as follows,
Case 1: The copy number and gene expression data for both groups of samples were generated with similar associational patterns.β^{G1} as well as β^{G2} are kept exactly the same, the value coming from a normal distribution with (mean for β^{G1}) = (mean for β^{G2}) $\neq 0$. Hence, there is no associational differences between groups of samples.
Case 2: The copy number and gene expression data for both groups of samples were generated with different associational patterns. For this, the copy number and gene expression data showed associations in $G1$ and no associations in $G2$. The values for β^{G1} are generated from normal distribution using a certain means and the data values for the samples with aberrations in $G1$ were generated using (1). Data values for all other samples in $G1$ and all samples in $G2$ were generated from a normal distribution with mean = 0 and variance = 1. Under each of these two cases we performed two simulation studies which can be described as follows,
Case 1.1 and Case 2.1: The distribution of copy number aberrations are kept exactly the same for the two groups of samples. Both groups consist of 50 samples each, with copy number aberrations in 25 randomly chosen samples for each group. The copy number aberrations are assigned in similar regions generated from normal distribution with (mean for $G1$) = (mean for $G2$) $\neq 0$. This makes sure there are no distributional differences between two groups for copy number aberrations.
Case 1.2 and Case 2.2: The distribution of copy number aberrations for one group of samples are made to differ from the other group. This is achieved by drawing the aberrations for the two groups from normal distribution with (mean for $G1$) $\neq$ (mean for $G2$) $\neq 0$. All the other parameters are kept exactly the same between simulation studies, including the number of samples, number of probes etcetera.

1.5 Sensitivity of dSIM towards changes in lambda values

We study the sensitivity of dSIM towards the variation of these λ values under two scenarios. First, we study the variation of λ value within and between different cases. For this, we perform four different simulation studies. In every simulation study, a certain parameter (number of probes, association differences, signal to noise ratio) is changed that affects the value of lambda.
Case 1 This case is used as the standard and all the other cases are compared to this case by changing on of the parameters (number of probes, associational patterns, etcetera). For this case, copy number and gene expression data are generated with 100 samples and 200 probes, with 50 samples in each group. Copy number aberrations are assigned to random samples and are generated

from normal distribution with (mean for $G1$) $\neq$ (mean for $G2$) $\neq 0$. Other samples with no copy number aberrations are assigned values generated from a normal distribution with mean = 0 and variance = 1. The gene expression data values for the aberrated samples in $G1$ are generated using (1) and β^{G1} is generated from normal distribution with a given mean. Gene expression data values for all other samples in $G1$ and all samples in $G2$ are generated from a normal distribution with mean = 0 and variance = 1.

Case 2 In this case we study the affect of changing the number of probes associated with gene expression. All the parameters are the same as in case one, except for the predefined stretch of probes for copy number aberrations. For this case, copy number and gene expression data are generated for 300 probes, instead of 200 as in Case 1.

Case 3 In this case, the associational patterns differ when compared to case 1. In case 1, only G1 showed associational patterns between copy number and gene expression. While, in this case $G1$ as well as $G2$ show associations between copy number and gene expression. The strengths of these associations differ between $G1$ and $G2$. This is achieved by drawing β^{G1} and β^{G2} from a normal distribution with (mean for β^{G1}) $\neq$ (mean for β^{G2}) $\neq 0$.

Case 4 For this case we vary the signal to noise ratio and then study the variation of lambda values when compared to case 1. All the parameters are kept same as in case 1 except the variance of the normal distribution. We increase the variance for generating copy number as well as gene expression data with more noise.

Second, we studied the effect of changing λ values over the dSIM p-values. For this we performed a simulation study where some simulated copy number probes showed differential association with gene expression data, while the others did not. The copy number data and gene expression data consisted of 50 samples and 20 probes, with 25 samples in each group. Out of these 25 samples, 70 percent in each group were selected and assigned values to a predefined stretch of probes from a normal distribution with mean $\neq 0$. The gene expression data values for the aberrated samples in $G1$ were generated using (1) and β^{G1} was generated with mean $\neq 0$. For this, a couple of probes were chosen out of the predefined region and only these probes showed differential association. For all other probes and samples, gene expression data values were generated from a normal distribution with mean = 0 and variance = 2.

2 dSIM test statistic

Let us define the model as

$$R_j = \delta c_j + \sum_{k=1}^{p} \gamma_k M_{jk} + \epsilon_j, \quad j = 1, \ldots, n. \tag{2}$$

where cross validated residuals $\{R_j\}$ are obtained as described in (3) in subsection 2.2 of the main text. The parameter of interest in this model is γ, which measures the differences in associations between the two groups of samples. Hence, in this model we want to test

$$H_0 : \gamma_k = 0, k = 1, \ldots, p \quad \text{against} \quad H_A : \gamma_k \neq 0, k = 1, \ldots, p$$

In the case when $p >> n$, there are alternatives which may lead to the same distribution of $\{R_j\}$ as H_0, meaning no power against these alternatives. One way of focusing the power of the test towards chosen interesting alternatives is to assign the vector $\boldsymbol{\gamma}$ a prior distribution. This will be similar to focusing the test against a set of chosen alternatives, making it possible for the test to gain more power against such alternatives.

If we assume that $\boldsymbol{\gamma}$ is a random variable with expectation zero and variance θ^2, then θ^2 determines how much the values in $\boldsymbol{\gamma}$ are allowed to deviate from zero. This changes the null and the alternative hypothesis to

$$H_0 : \theta^2 = 0 \quad \text{against} \quad H_A : \theta^2 \neq 0.$$

Now suppose that $\boldsymbol{\gamma} = \theta\mathbf{b}$, where $E(\mathbf{b}) = 0$ and $E(\mathbf{b}\mathbf{b}^\top) = \Sigma$ where Σ is a positive definite matrix and the distribution of $\mathbf{b}$ does not depend on θ. With the assumption that the log-likelihood $log\{f(\boldsymbol{\gamma}; R)\}$ and its first two derivatives exist, we can define the score test as

$$S = \frac{1}{2}\mathbf{s}^\top\Sigma\mathbf{s} - \frac{1}{2}trace(\Sigma\mathbf{I})$$

where $\mathbf{s}$ is the score function defined as $\mathbf{s} = \frac{\partial}{\partial\gamma}log\{f(0, R)\}$ and $\mathbf{I}$ is the observed Fisher information given as $\mathbf{I} = -\frac{\partial^2}{\partial\gamma\partial\gamma^\top}log\{f(0, R)\}$.

In a linear model setting such as the one given in (2), we can assume $R \sim \mathcal{N}(\gamma\mathbf{M}, \sigma^2 I)$ for defining the score vector as $\mathbf{s} = (\sigma^{-2}\mathbf{M}^\top R)$ and the Fisher information as $\mathbf{I} = (\sigma^{-2}\mathbf{M}^\top\mathbf{M})$. This gives the general score test statistic as,

$$S^*_\Sigma = \frac{1}{2\sigma^4}R^\top\mathbf{M}\Sigma\mathbf{M}^\top R - \frac{1}{2\sigma^2}trace(\mathbf{M}\Sigma\mathbf{M}^\top).$$

Since σ^2 is not known we can replace it with its maximum likelihood estimate under the null hypothesis, leading to the test statistic,

$$S_\Sigma = \frac{R^\top\mathbf{M}\Sigma\mathbf{M}^\top R}{R^\top R},$$

whose distribution does not depend on σ^2. By assuming $\Sigma = I$, we can get the locally most powerful test given as

$$S = \frac{R^\top\mathbf{M}\mathbf{M}^\top R}{R^\top R}.$$

3 Table S1

This table gives the selected copy number probes and gene expression probes on chromosome arm 12p for NKI data. We selected 5 copy number probes with significant dSIM p.values. For a selected copy number probe, we started with choosing only the significantly associated gene expression probes. This selection was based on the individual global test p-values between the copy number probe and every gene expression probe (global test p-value < 0.1). The group of significantly associated gene expression probes were then arranged in the order of increasing difference between their genomic locations and that of the copy number probe. The top four gene expression probes were then selected from this group based on their proximity.

Copy number probe 1	
ID	RP5-1096D14_2107_1
start position	1855112

Gene expression probes arranged in increasing genomic location differences from copy number probe 1			
IDs	absolute start position	globaltest pvalues	increasing genomic location differences
Contig3422_RC	1797740	0.08	57372
NM_002879	1021243	0.01	833869
Contig57181	861759	0.05	993353
NM_018463	2921788	0.08	1066676
Contig30267_RC	2986389	0.02	1131277
Contig46324_RC	2986389	0.04	1131277
NM_003324	2986389	0.05	1131277
NM_003213	3068496	0.08	1213384
AL133026	389223	0.08	1465889
NM_016615	329789	0.09	1525323
NM_020375	4430371	0.04	2575259
Contig2524_RC	4596894	0.10	2741782
Contig57584_RC	6957967	0.07	5102855
NM_003481	6961292	0.00	5106180
NM_001940	7033626	0.04	5178514
NM_007273	7074490	0.00	5219378
NM_016546	7242183	0.01	5387071
NM_014718	7282294	0.01	5427182
NM_000319	7341281	0.00	5486169
NM_004244	7623409	0.04	5768297
NM_015509	7926148	0.02	6071036

Copy number probe 2	
ID	RP11-277E18_2115_1
start position	7918138

Gene expression probes arranged in increasing genomic location differences from copy number probe 2			
IDs	absolute start position	globaltest pvalues	increasing genomic location differences
NM_015509	7926148	0.04	8010
NM_000319	7341281	0.00	576857
NM_014718	7282294	0.05	635844
NM_007273	7074490	0.00	843648
NM_001940	7033626	0.05	884512
NM_003481	6961292	0.00	956846
Contig2524_RC	4596894	0.01	3321244
NM_003213	3068496	0.00	4849642
Contig46324_RC	2986389	0.00	4931749
Contig30267_RC	2986389	0.01	4931749
NM_003324	2986389	0.03	4931749
NM_018463	2921788	0.01	4996350
NM_002879	1021243	0.01	6896895
Contig57181	861759	0.00	7056379
AL133026	389223	0.00	7528915

Copy number probe 3	
ID	RP11-436I9_2117_1
start position	9067838

Gene expression probes arranged in increasing genomic location differences from copy number probe 3			
IDs	absolute start position	globaltest pvalues	increasing genomic location differences
NM_000319	7341281	0.00	1726557
NM_007273	7074490	0.01	1993348
NM_001940	7033626	0.01	2034212
NM_003481	6961292	0.00	2106546
NM_003213	3068496	0.00	5999342
Contig46324_RC	2986389	0.00	6081449
Contig30267_RC	2986389	0.00	6081449
NM_003324	2986389	0.00	6081449
NM_018463	2921788	0.05	6146050
NM_002879	1021243	0.00	8046595
Contig57181	861759	0.02	8206079
AL133026	389223	0.00	8678615

Copy number probe 4	
ID	RP11-320J20_2124_1
start position	15496292

Gene expression probes arranged in increasing genomic location differences from copy number probe 4			
IDs	absolute start position	globaltest pvalues	increasing genomic location differences
NM_000319	7341281	0.03	5430844
NM_007273	7074490	0.07	5697635
NM_001940	7033626	0.05	5738499
NM_003481	6961292	0.01	5810833
NM_003213	3068496	0.09	9703629
Contig46324_RC	2986389	0.00	9785736
Contig30267_RC	2986389	0.01	9785736
NM_003324	2986389	0.02	9785736
NM_002879	1021243	0.02	11750882
AL133026	389223	0.01	12382902

Copy number probe 5	
ID	RP11-328P13_2128_1
start position	19403674

Gene expression probes arranged in increasing genomic location differences from copy number probe 5			
IDs	absolute start position	globaltest pvalues	increasing genomic location differences
NM_000319	7341281	0.06	8155011
NM_007273	7074490	0.03	8421802
NM_001940	7033626	0.03	8462666
NM_003481	6961292	0.01	8535000
NM_003213	3068496	0.07	12427796
Contig46324_RC	2986389	0.00	12509903
NM_003324	2986389	0.00	12509903
Contig30267_RC	2986389	0.01	12509903
NM_002879	1021243	0.02	14475049
AL133026	389223	0.00	15107069

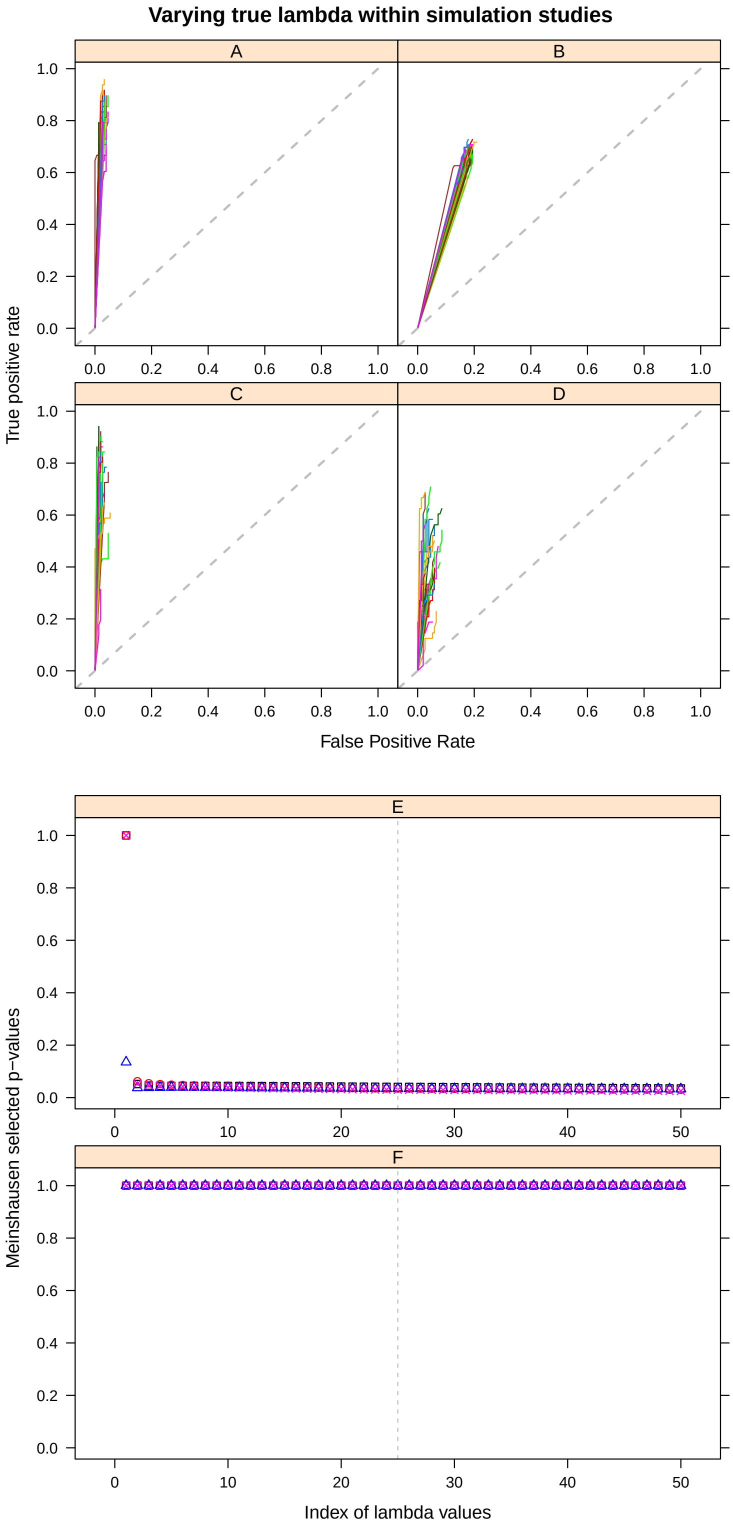

Figure S1: **(A)**:ROC curve for simulation study 1, 200 probes. **(B)**: ROC curve for simulation study 2, 300 probes. **C)**: ROC curve for simulation study 3, different association differences, 200 probes. **(D)**: ROC curve for simulation study 4, higher signal to noise ratio, 200 probes. **(E)**: Meinshausen selected p-values for probes with differential association on a range of lambda values. The colored triangles, squares, circles and crosses depict the p-values for probes which show differential associations in the simulation study and are selected by dSIM as significant (true positives). Grey dotted line marks the index for λ_{opt} **(F)**: Meinshausen selected p-values for probes with no differential association on a range of lambda values. The colored triangles, squares, circles and crosses depict the p-values for probes which do not show differential associations in the simulation study and are not selected by dSIM as significant (true negatives). Grey dotted line marks the index for λ_{opt}

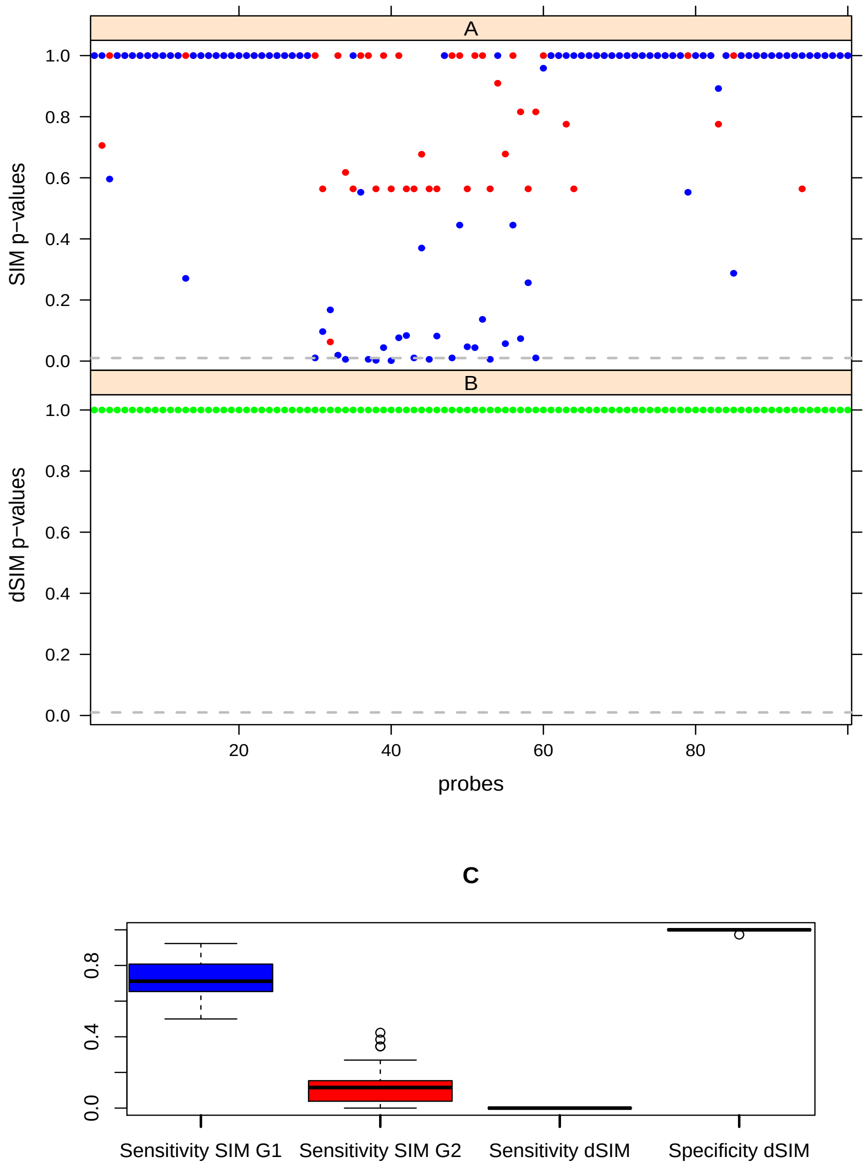

Figure S2: **(A)**: Separate analysis (SIM) detection for group 1 samples (blue dots) and group 2 samples (red dots). **(B)**: Meinshausen selected dSIM p.values. **C)**: Sensitivity of separate analysis towards the larger group 1 (G1, blue boxplot), smaller group 2 (G2, red boxplot) and sensitivity and specificity for dSIM.

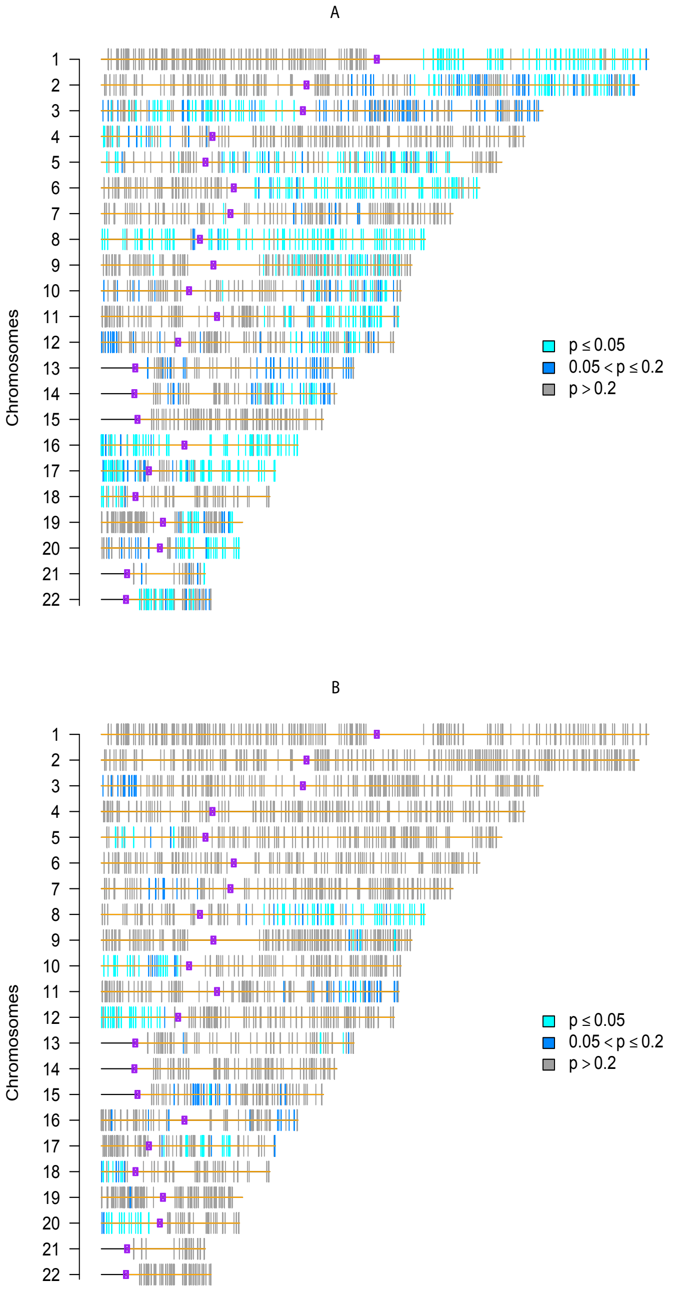

Figure S3: **(A)**: Probes selected from separate analyses for ER positive samples (43), NKI breast cancer data. **(B)**: Probes selected from separate analyses for ER negative samples (25), NKI breast cancer data. Chromosomes are represented by horizontal bars. Each vertical bar represents one copy number probe, with color of the bar indicating the test result: blue, significant (FDR < 0.05); gray, not significant (FDR > 0.05).

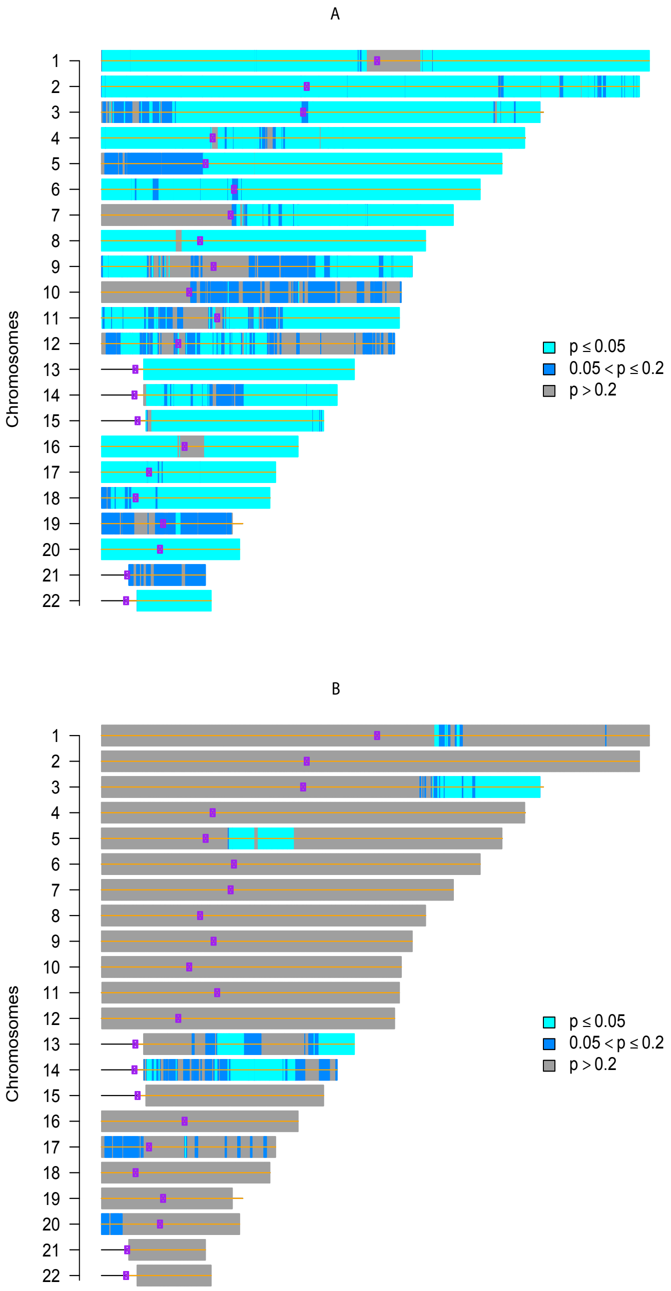

Figure S4: **(A)**: Probes selected from separate analyses for ER positive samples (134), TCGA breast cancer data. **(B)**: Probes selected from separate analyses for ER negative samples (32), TCGA breast cancer data. Chromosomes are represented by horizontal bars. Each vertical bar represents one copy number probe, with color of the bar indicating the test result: blue, significant (FDR < 0.05); gray, not significant (FDR > 0.05).

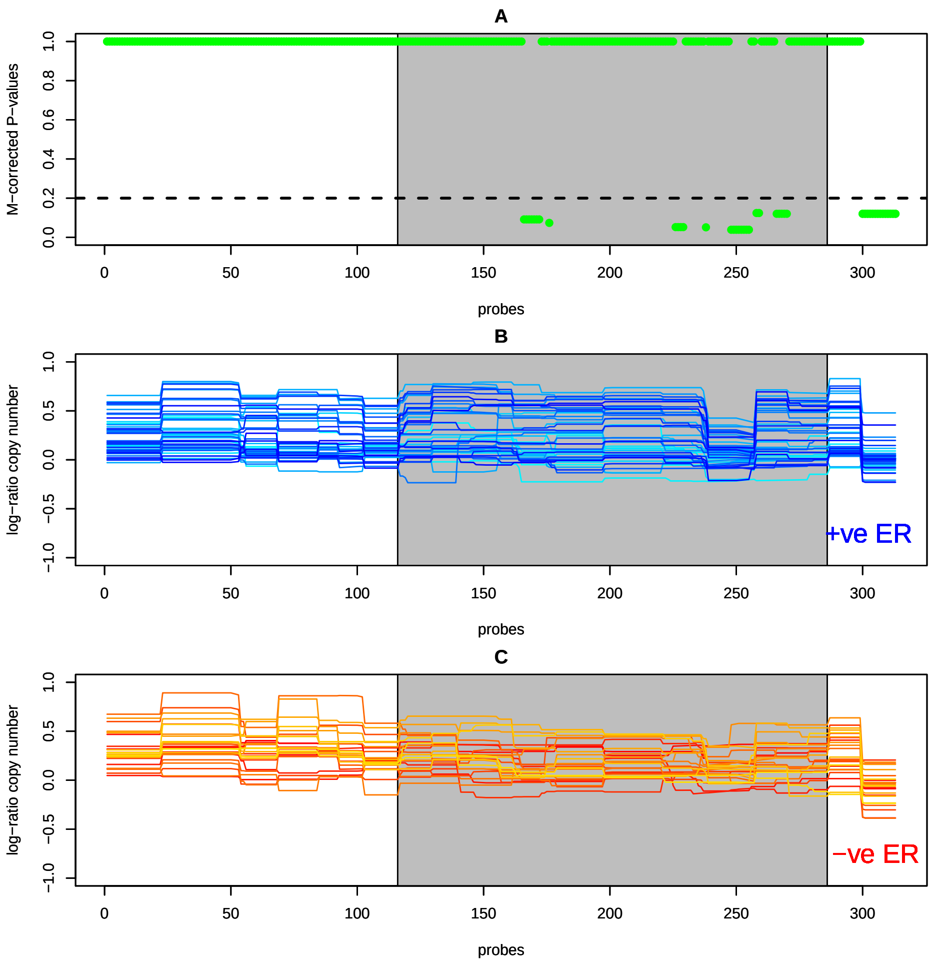

Figure S5: **(A)**: Selected probes by dSIM for NKI breast cancer data on chromosome arm 1q. **(B)**: Copy number profile for ER positive samples (43), chromosome arm 1q. **(C)**: Copy number profile for ER negative samples (25), chromosome arm 1q. Here the x-axis scale used places probes equally spaced, rather than using their genomic location, to facilitate visualization of copy number change. In terms of genomic position, the gray region extends from 157.1 Mb to 240 Mb.

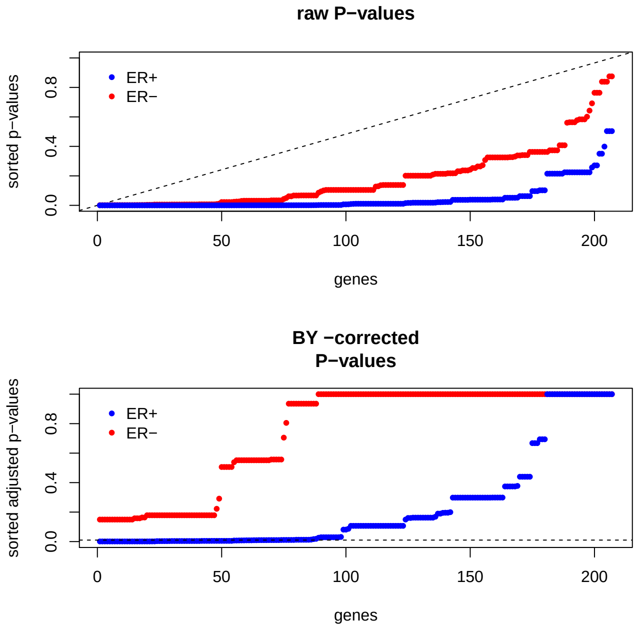

Figure S6: **A)**: Distribution of raw p-values obtained from separate analyses for chromosome arm 3p, ER positive group (blue color,43 samples) and ER negative group (red color,25 samples), NKI dataset. **(B)**: Distribution of Benjamini-Yekutieli adjusted p-values obtained from separate analyses for chromosome arm 3p, ER positive group (blue color,43 samples) and ER negative group (red color,25 samples), NKI dataset.

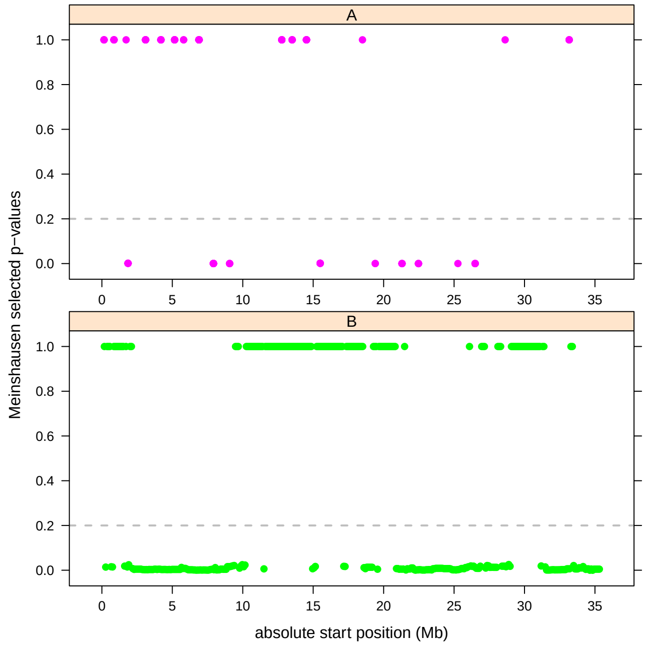

Figure S7: **(A)**: Probes selected by dSIM on 12p for NKI (68 samples) breast cancer data. **(B)**: Probes selected by dSIM on 12p for TCGA (166 samples) breast cancer data.

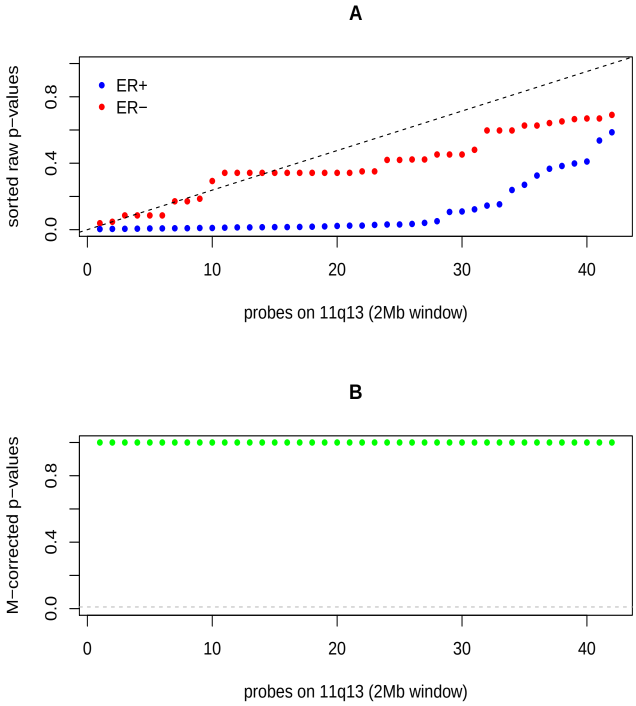

Figure S8: **(A)**:Distribution of raw p-values obtained from separate analyses for chromosome region 11q13, ER positive group (blue color,134 samples) and ER negative group (red color,32 samples), TCGA dataset. **(B)**: Meinshausen selected dSIM p-values for 11q13 chromosome region. The horizontal dotted grey line represents the cutoff level.

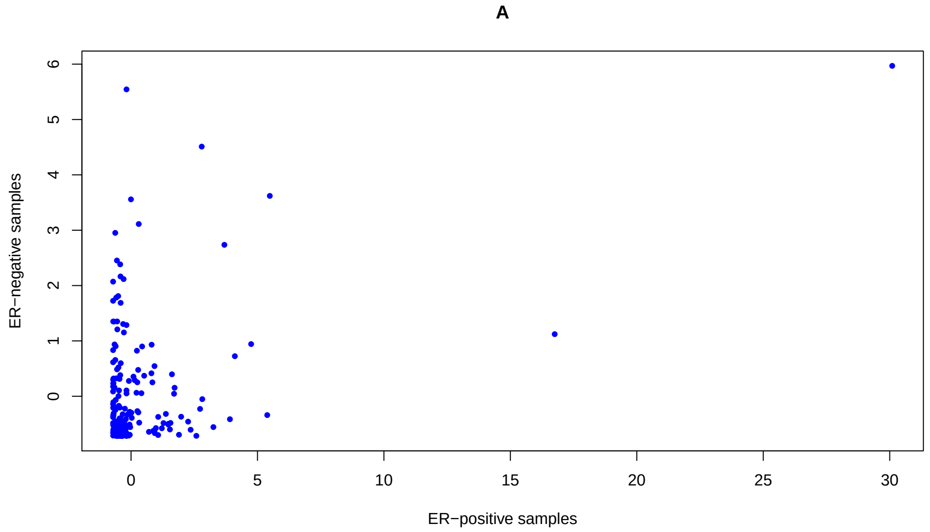

Figure S9: **(A)**: Scatter plot of global test z-scores for ER positive and ER negative samples.

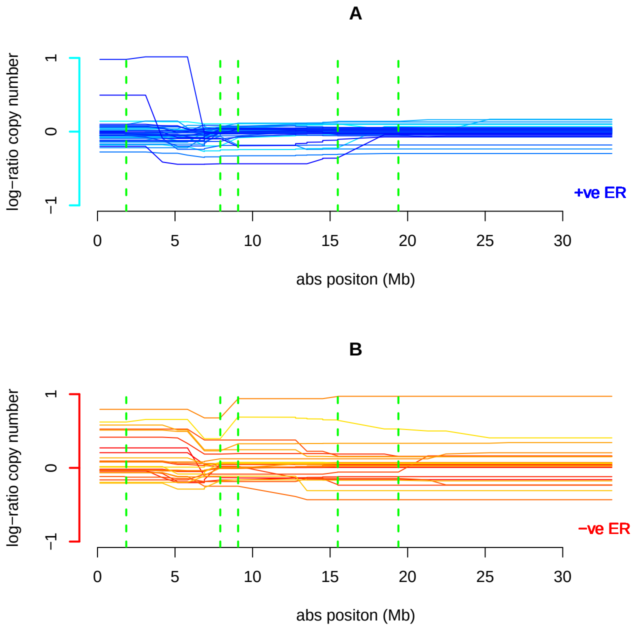

Figure S10: **(A)**: Copy number probes (green dotted bars) selected by dSIM on 12p for ER positive samples (43) in NKI breast cancer data. **(B)**: Copy number probes (green dotted bars) selected by dSIM on 12p for ER negative samples (25) in NKI breast cancer data.

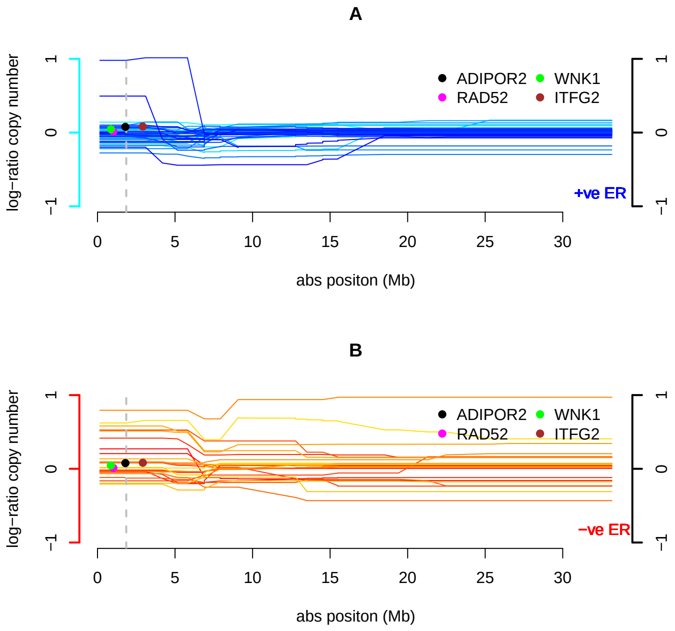

Figure S11: **(A)**: Significant gene expression probes (colored dots) selected for copy number probe (grey dotted line) on 12p for ER positive samples (43) in NKI breast cancer data. **(B)**: Significant gene expression probes (colored dots) selected for copy number probe (grey dotted line) on 12p for ER negative samples (25) in NKI breast cancer data.

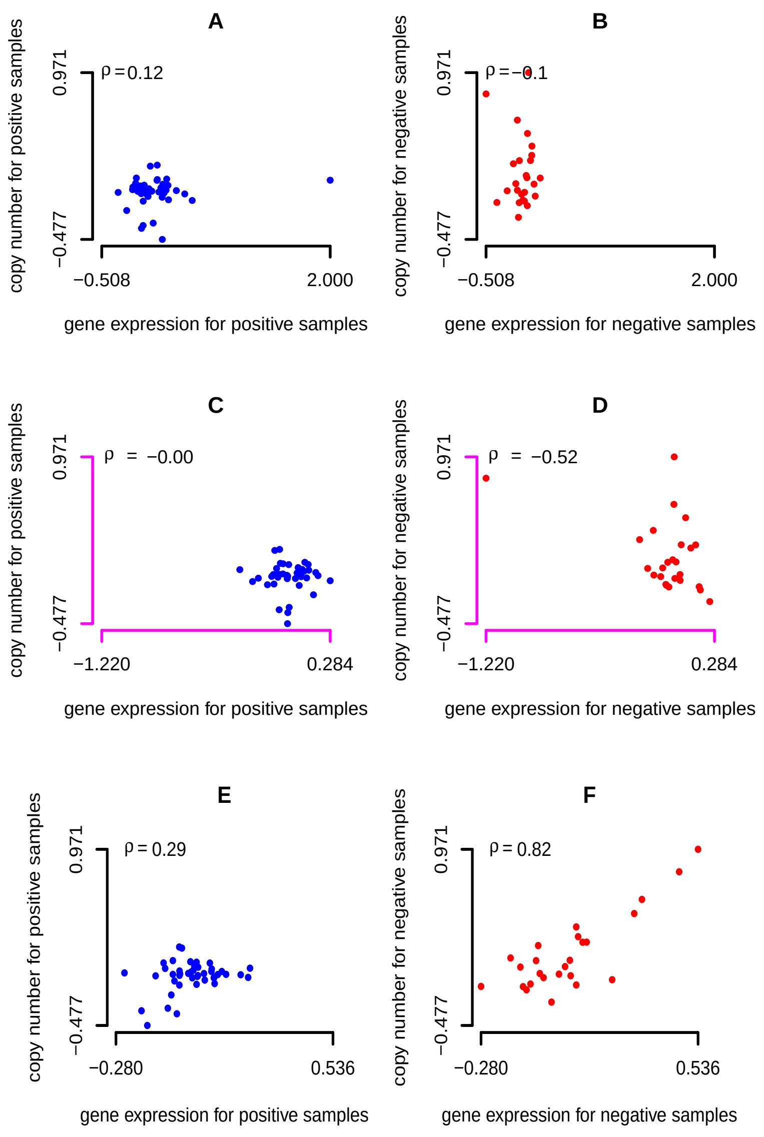

Figure S12: **(A)**: Association between selected copy number probe and gene expression probe ($NANOG$) data points for all ER positive samples. **(B)**: Association between selected copy number probe and gene expression probe ($NANOG$) data points for all ER positive samples. **(C)**: Association between selected copy number probe and gene expression probe ($GDF3$) data points for all ER positive samples. **(D)**: Association between selected copy number probe and gene expression probe ($GDF3$) data points for all ER negative samples. **(E)**: Association between selected copy number probe and gene expression probe ($PEX5$) data points for all ER negative samples. **(F)**: Association between selected copy number probe and gene expression probe ($PEX5$) data points for all ER negative samples.
